# Introduction of hospital-based health technology assessment in China: experiences from seven pilot hospitals

**DOI:** 10.1017/S0266462323002738

**Published:** 2024-01-29

**Authors:** Lanting Lyu, Wenkai Shi, Kristian Kidholm, Fei Bai, Xia Lin, Jinlan Fu, Tianqing Li, Guoxun Li, Li Luo, Ting Wang, Hai Yang

**Affiliations:** 1 School of Public Administration and Policy, Renmin University of China, Beijing, China; 2 Health Technology Assessment and Policy Evaluation Group, Renmin University of China, Beijing, China; 3 Chinese Academy of Fiscal Sciences, Beijing, China; 4 Center for Innovative Medical Technology, Odense University Hospital, Odense, Denmark; 5 National Center for Medical Service Administration, National Health Commission of the People’s Republic of China, Beijing, China; 6 National Center for Cardiovascular Diseases, Beijing, China; 7 Medical Equipment Information Implementation Practice and Training Center, China–Japan Friendship Hospital, Beijing, China; 8 People’s Hospital of Tianjin, Tianjin, China; 9 Sixth People’s Hospital Affiliated of Shanghai Jiao Tong University, Shanghai, China; 10 Medical Administration Department, Beijing Chao-Yang Hospital, Beijing, China

**Keywords:** hospital-based health technology assessment, hospital management, case study, decision making, China, health reform

## Abstract

**Objectives:**

This study aimed to introduce a pilot program for hospital-based health technology assessment (HB-HTA) in China and present the participants’ experiences based on seven case studies from seven tertiary hospitals.

**Methods:**

One-year pilot projects were initiated at the beginning of 2018. Seven pilot hospitals were closely followed from the beginning until the completion of their pilot HTA project. Regular interviews were conducted with the hospital managers leading the HB-HTA projects and key members of the special HTA teams. Observations were made based on field trips and written HTA reports.

**Results:**

Three pilot projects evaluated the use of medical consumables, three evaluated the use of surgical or medical interventions, and one evaluated an innovative management model for ventilators. Real-world data were collected from all the pilot projects to assist with the assessments. Most HB-HTA pilot projects achieved remarkable results such as improvements in economic efficiency; however, there were also obvious deficiencies such as the lack of a necessary cost-effectiveness analysis.

**Conclusions:**

The results varied among the seven HB-HTA pilot projects. The HB-HTA pilot program was implemented to promote the use of HB-HTA in hospital decision making in China. At the same time, HB-HTA in China faces challenges. We have made some policy recommendations based on the findings of the pilot projects.

## Introduction

Health technology assessment (HTA) is a comprehensive method for evaluating the technical performance and economic characteristics of medical equipment and devices, diagnosis and treatment technologies, and drugs. It is an indispensable tool in health technology decision making and management (1;2). In recent years, HTA policy frameworks have been widely applied in Asian countries and regions (3). Even though HTA originally focused on decision making on a national or regional level, the need and demand for the implementation of hospital-based health technology assessment (HB-HTA) for hospital-level decision making has increased (4). The speed of innovation in health technology is higher than ever, and hospitals are faced with numerous decisions on whether to invest in new health technologies. These health technologies can include the acquisition, implementation, or discontinuation of new or out-of-date technologies, interventions, and management/operational changes that require investment (or disinvestment in the case of discontinuation). Simultaneously, hospitals must comply with regulations and sometimes health insurance-related contract conditions. Tertiary hospitals in China may receive 80–150 applications for new health technologies per year based on information gathered from the interviews conducted in this study.

As a scientific tool, HB-HTA is applied to facilitate the production of a basis for decision making within hospitals, hospital groups, or coalitions (5). Hospitals, as the primary entities in charge of the administration of clinical applications of medical technologies, are generally the entry point for many new technologies in China. In the real world, where resources are limited, patient demand to be treated with the most innovative technologies is growing. Thus, hospitals worldwide are forced to develop their own HB-HTA systems to guide them in investment or disinvestment decision making based on their actual needs (6;7). Therefore, hospitals must have the necessary capabilities for HB-HTA before they can use the tool in a scientifically valid manner (8).

The European Adopting Hospital-based Health Technology Assessment (AdHopHTA) project has produced useful guidelines on HB-HTA (9–11). The establishment of an HB-HTA unit in a hospital builds capacity and creates a basis for making informed managerial decisions, identifying key directions for strategic development, and improving hospital management (12;13).

Some of China’s large tertiary hospitals with strong research branches are in the early stages of conducting exploratory HB-HTA activities; however, most hospitals are yet to apply HB-HTA and may have specific problems in initiating such work. In 2009, China launched a new round of health reforms focusing on four aspects: public health service, medical service, medical security, and drug supply security systems. China’s health reforms have made hospitals themselves, rather than local health departments, responsible for accessing health technology, medical services, procurement, and management since November 2018. Simultaneously, hospitals face restrictions mainly set by two national organizations: the National Health Commission and the National Healthcare Security Administration. To promote HB-HTA and ensure that it plays a greater role in providing evidence-based decision making and to achieve better management and higher quality in services, HB-HTA pilot programs were initiated by the authors’ institute.

This study describes HB-HTA pilot programs in China and presents participants’ experiences based on all seven case studies. Thus, the study shares valuable experiences that will not only help China to promote the application of HB-HTA in the future but may also inspire other countries.

## Methods

The National Center for Medical Service Administration (NCMSA) of the National Health Commission of China launched the first batch of HB-HTA pilot research projects in March 2018, with seven pilot hospitals trying out a self-selected HB-HTA project. In March 2019, a second batch of pilot research projects was launched in which 23 hospitals were selected as pilot research units. The details of the first batch of HB-HTA pilot hospitals are presented in Table 1. To ensure a smoother execution of the pilot project, hospitals with more than 1,500 beds, known as 3A hospitals, were selected since they have stronger technical, financial, and personnel resources to carry out HB-HTA work.

**Table 1.** The first batch of HB-HTA pilot hospitals in China

Name	Location	Type and grade	Health technology type	Parameters	The composition of the HTA team
The Sixth People’s Hospital of Shanghai	Shanghai	3A, General Hospital	HTA of hANG (a new material) for patients need peripheral nerve defect repair	Safety, effectiveness, economic	Experts of HB-HTA interest group, orthopedic emergency group, and manufacturer technicians. Members with the background of health policy research and health economics conducted an internal review during the evaluation process, and researchers in the HB-HTA interest group conducted an external review
West China Hospital of Sichuan University	Chengdu, Sichuan	3A, General Hospital	HTA on clinical application of innovative suture in surgery	Safety, effectiveness	Experts of HB-HTA interest group, the deputy director of the hospital, statistician with trainings of evidence-based medicine within the hospital, and surgeons in the hospital. Members with the background of EBM and health economics conducted an internal review during the evaluation process, and researchers in the HB-HTA interest group conducted an external review
Guangzhou Women and Children’s Medical Center	Guangzhou, Guangdong	3A, Women and Children’s Hospital	HTA on suture based on Information Technology	Safety, effectiveness, economy	Clinical experts, manufacturers, and suppliers inside and outside the hospital conducted the review
QILU Hospital of Shandong University	Jinan, Shandong	3A, General Hospital	HTA on transcatheter aortic valve replacement	Safety, effectiveness, economy	Experts of HB-HTA interest group, the director of hospital information department, clinical experts on cardiology, and cardiac surgery in the hospital. Members with the background of cardiology and cardiac surgery and health economics conducted an internal review during the evaluation process, and researchers in the HB-HTA interest group conducted an external review
The First Hospital of Jilin University	Changchun, Jilin	3A, General Hospital	HTA on haploid transformation in the treatment of aplastic anemia	Safety, effectiveness, economy, organization; Patients’ feelings; Strategic value	Experts from relevant administrative departments and clinical departments, not involving drug and device manufacturers conducted the review
People’s Hospital of Tianjin	Tianjin	3A, General Hospital	HTA of fecal flora transplantation	Safety, effectiveness	Experts of HB-HTA interest group, the hospital manager in charge, and the director of gastroenterology department of the hospital. Members with the background of gastroenterology and health economics an conducted internal review during the evaluation process, and researchers in the HB-HTA interest group conducted an external review
The China-Japan Friendship Hospital	Beijing	3A, General Hospital	HTA of a management model for ventilators	Safety, effectiveness, economic, and social effects	Experts of HB-HTA interest group, the hospital manager in charge, the hospital equipment department, and manufacturer technicians. Members with the background of equipment management and health economics conducted an internal review during the evaluation process, and researchers in the HB-HTA interest group conducted an external review

HB-HTA, hospital-based health technology assessment.

A team of HTA researchers, organized by the authors’ institute, provided a three-day training program for all personnel involved in the project. Templates for HTA reports and handbooks – adapted from the AdHopHTA handbook – were produced by the author’s organization (14) and issued by the authors’ institute at the end of each training session. The handbook was translated into Chinese, and the templates in the handbook were used in the pilot projects. Additional explanations were also added to the templates. The first pilot courses included an outline of HTA, process and basic methods, methods for conducting systematic reviews, and methods for economic evaluations. Based on the feedback, a half-day course with a hands-on session was added to the training for the second pilot. For the individual HTA pilot projects, we advised all trainees to learn from the AdHopHTA handbook. An advisory team was available to help if/when they had technical difficulties. No rules were set for participating hospitals on what to evaluate. The first seven hospitals all completed their chosen HTA pilot project, and HTA reports were submitted after 8 months.

To collect information about participants’ experiences in the production of the HB-HTA reports, we interviewed all seven participating teams to collect their feedback. Two researchers interviewed key personnel in charge of the HB-HTA pilot program. In each pilot hospital, we chose 1–2 key individuals in charge of the pilot HTA project as interviewees; a total of 10 interviewees were chosen. The interviews were conducted according to the semi-structured interview outline shown in Table 2. The interview time for each interviewee was about 30 min, and a specially assigned person was responsible for the recording. After the interviews, we used the Colaizzi 7-step analysis method to classify and summarize the key factors affecting the implementation of the first batch of HB-HTA pilot projects in China (this process was published in another paper) (15).

**Table 2.** Questions of the interview with key informants in the pilot hospitals

Background	Q1: What is the health technology evaluated in the HB-HTA pilot?
	Q2: Why does your hospital choose this technology for the pilot tryout?
Process	Q3: What is the detailed process of carrying out HB-HTA in hospitals?
	Q4: Who participated in the actual assessment?
Results	Q5: What are the results of HB-HTA in hospitals? (technology, safety, clinical effectiveness, ethics and law aspects, economic aspects, organizational aspects, social and public policy aspects, and hospital strategic aspects)
Conclusion and recommendations	Q6: What are the problems or deficiencies of HB-HTA in hospitals?
	Q7: What are the achievements of HB-HTA in hospitals?
	Q8: What are the suggestions for the future development of HB-HTA in your hospital?
	Q9: What are your suggestions for other hospitals to carry out assessment of technology in the future?

HB-HTA, hospital-based health technology assessment.

In this article we focus on seven cases from pilot hospitals: (i) the HTA of medical consumables for the Sixth People’s Hospital of Shanghai, West China Hospital of Sichuan University, and Guangzhou Women and Children’s Medical Center; (ii) the HTA of a new medical technology for the QILU Hospital of Shandong University, the First Hospital of Jilin University, and People’s Hospital of Tianjin; and (iii) the HTA of an innovative management model for ventilators for the China–Japan Friendship Hospital in Beijing.

## Results

### Case 1: HB-HTA of Medical Consumables Management in the Sixth People’s Hospital of Shanghai

#### Background of the Hospital’s HB-HTA Project

Implantable medical devices are an important category of medical consumables because of their high unit price and relatively complex technology, which has been the focus in China’s public hospitals given the difficulty related to procurement management. The authors’ institute has a strong focus on orthopedics and neurosurgery, in which many high-value medical consumables (in orthopedics and neurosurgery) are used. The Sixth People’s Hospital of Shanghai chose acellular allogeneic nerve repair material as an intervention in the HTA pilot project. Patients included those who needed treatment to repair peripheral nerve defects. The comparators included direct suturing, autologous nerve transplantation, nerve catheter transplantation (mainly using non-biological materials), and allogeneic material (hANG, a new and relatively expensive material) transplantation. Outcomes were measured using the Static Two-Point Discrimination (S2-PD) form for sensory nerve recovery and economic burden on patients was also estimated.

#### HB-HTA of hANG for Patients Needing Peripheral Nerve Defect Repair

The HTA mainly involved the hospital’s HB-HTA team. The team leader was an experienced manager with HTA training. Five team members across three management departments were involved, all of whom were postgraduates with majors in biomedical engineering, hospital management, health policy, and health economics. All team members had some prior HTA training before the HTA training program for the pilot projects. In addition, clinical experts, including the head of the orthopedic emergency group and technical personnel of the manufacturer, were involved in the production of the HTA. The HB-HTA team was responsible for the evaluation. External reviewers were invited to review the HTA reports; however, patients were not included.

**Figure 1.**
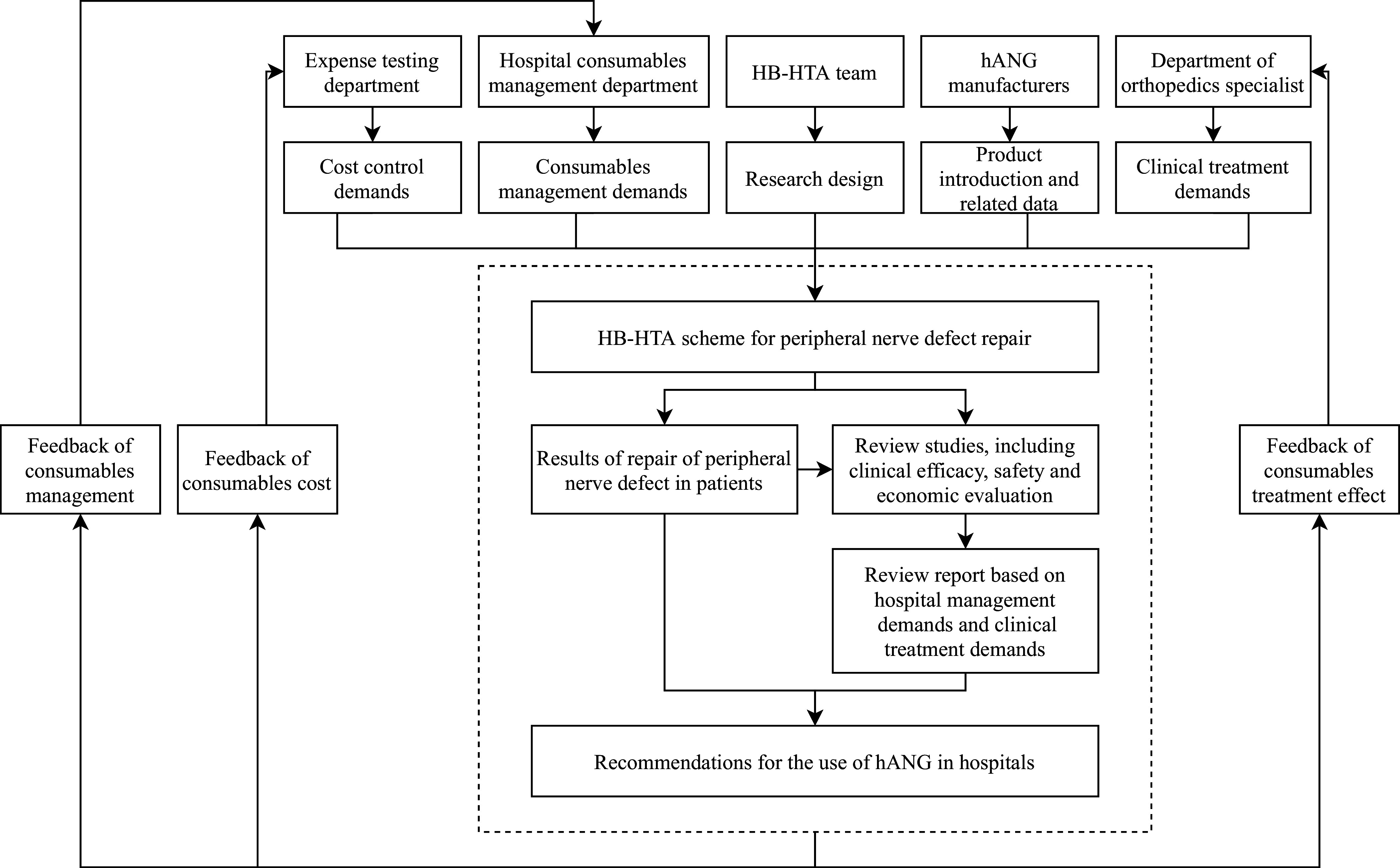
Technical roadmap for hospital-based health technology assessment (HB-HTA) of peripheral nerve defect repair materials in the Sixth People’s Hospital of Shanghai.

First, the team conducted a literature review to obtain peer-reviewed information. A total of 34 articles were included in the literature review. No relevant HTA or economic evaluation study was found; therefore, the team decided to collect hospital-based data. Clinical and economic data of patients’ costs were collected from the clinic department using a survey form, in addition to extracting data from the Hospital Information System (HIS). HTA was then carried out on a step-by-step basis using the template introduced by the authors’ institute and the European AdHopHTA. In the later stages of the assessment, three researchers from scientific research institutions and universities were invited to conduct a quick external review for quality assurance. The HTA draft report was sent to external reviewers and a face-to-face meeting was arranged to collect feedback and suggestions for improving the HTA. A technical roadmap for the HB-HTA for peripheral nerve defect repair materials is shown in Figure 1.

Data on the efficacy and safety of peripheral nerve defect repair were obtained from the literature and HTA databases. The clinical effectiveness and costs for all patients who received treatment for peripheral nerve defect repair were collected and recorded, including the usage of allogeneic materials, patient characteristics, outcomes, costs, and other related surgeries. Relevant data were collected from the internal HIS of the pilot hospital.

Based on the assessment results using information from the literature and data from the HIS, the HB-HTA team concluded that hANG has clinical advantages compared with traditional technology using other medical consumables, but only when the length of the patient’s nerve defect exceeds 12 mm. In other words, the HTA report recommended the restricted use of hANG in clinical practice. The in-house access committee for medical technology accepted the HTA report’s recommendations. After the implementation of the committee’s recommendation for clinical practice, the use of hANG was reduced by almost a half within a month. The average cost of hANG per surgery is significantly higher (about 2.4 times) than that of nerve conduits. In 2017, hospitals used 672 hANG, which accounted for 42.40 percent of all nerve repair materials used; moreover, valued at 13.18 million RMB, hANG accounted for 64 percent of total costs on nerve repair materials.

#### Lessons Learned from this Case

The Sixth People’s Hospital of Shanghai has a relatively long history of applying HB-HTA in decision making and conducted the first HTA in 2008. After interviewing key persons in the production and use of this HB-HTA, the main reasons for its success can be summarized as follows. First, and most importantly, hospital management was willing to use scientific management tools such as HTA as the basis for decision making. Second, the hospital established an HTA working group with HTA skills that was responsible for the HTA project’s management and evaluation. This ensured the scientific and professional aspects of the assessment activities. Third, changes in clinical practice were made along with the assessment. The clinical lead, who was involved in the assessment right from the start, immediately initiated assessment-relevant changes in clinical practice when new and important evidence was discovered. The hospital’s HB-HTA team relied mainly on literature reviews for better evidence and critically considered the information provided by the manufacturer. Fourth, the use of real-world data (RWD) is crucial for HTA (16), especially in China, owing to the lack of routinely collected data that fit the purpose of HTA. However, RWD also have limitations, as RWD are the results of a mixture of confounding factors that may or may not have an impact over the results of interest. In general, effective access to RWD is still weak in all hospitals in China. The hospital provided strong support for evaluation activities by collecting the necessary data straight from the hospital information database and conducting a survey in hospital settings, which was helpful to compare both the costs and effectiveness of different medical consumables.

### Case 2: HB-HTA Pilot Project at the West China Hospital of Sichuan University

#### Background of the HB-HTA Project

The West China Hospital of Sichuan University is home to the first Cochrane China Center and has therefore fostered a culture of evidence-based medicine research and decision making. Thus, the hospital has the necessary professional foundation to carry out HB-HTA. At the start of the HB-HTA pilot, HTA at West China Hospital was still in a very early stage of implementation. Therefore, the team at the hospital chose to evaluate a medical consumable, barbed sutures, as the HB-HTA pilot, but they also emphasized using this pilot project to explore the general process of HB-HTA for expanding the HB-HTA implementation as a research question and in hospital real-world decision making.

#### Research on HB-HTA and the Example of Barbed Sutures

First, an HB-HTA team was built, and one research fellow from the Cochrane China Center was appointed to manage the pilot, while the hospital’s deputy chief responsible for medical affairs supervised the pilot. The HB-HTA team then gathered information from hospital administrators, physicians, and evidence-based medicine experts to understand the current process of introducing new consumables into the hospital, and the HTA knowledge and skills they may have. They also conducted a literature review that was within the team’s skill set. A comprehensive literature review on the safety and efficacy of barbed sutures in operations was comprehensively compiled and analyzed. However, the team did not evaluate the economic aspects for the use of barbed sutures.

#### HTA Results

In the assessment of barbed sutures, West China Hospital summarized in detail the effectiveness and safety of barbed sutures in four types of procedures by systematically analyzing 25 studies in the literature, seven randomized controlled trials (RCTs), and 21 cohort studies. The systematic reviews were carried out with excellence; however, the pilot project did not conduct an economic analysis of the selected consumable.

#### Lessons Learned from this Case

West China Hospital was proactive in first studying its current management for the introduction of consumables and the standard HTA process, which provided a reference for the later establishment of its HB-HTA mechanism. In the analysis of barbed sutures, the hospital showed its excellent evidence-based medicine skills and fully demonstrated the effectiveness and safety of the consumable but without including an economic dimension. The team has a very strong research background in the field of evidence-based medicine, but none in the field of economic evaluation of health technologies. As a result, it was difficult to incorporate an economic analysis into the pilot evaluations. Thus, training is key to the success of a full HB-HTA. In addition, to promote HB-HTA, a rigorous mechanism needs to be established and all aspects of HTA should be considered before making decisions.

### Case 3: HB-HTA of Sutures Based on a Specifically Designed Information System in Guangzhou Women and Children’s Medical Center

#### Background of the Hospital’s HB-HTA Project

Medical consumption management is an important part of hospital health technology management. In recent years, China has implemented a series of payment reforms such as “zero mark-up” for medical consumables and bulk purchases of medical consumables to control the rapid rise of costs associated with using medical consumables. At a hospital level, the sophisticated management of consumables has become an important aspect of healthcare technology management. The Guangzhou Women and Children’s Medical Center (GWCMC) explored the assessment of sutures based on a specifically designed information system, which is also a database of information of medical consumables that is useful for the selection prior-decision, the procurements and the control of consumables usage.

#### HB-HTA Process and Results

The HB-HTA group at GWCMC had prior experience of HB-HTA and had applied the methods in real-world decision making, although they did not follow the AdhopHTA framework. GWCMC’s HB-HTA project mainly evaluated four aspects: safety, effectiveness, economics, and cooperation. GWCMC’s pilot was to establish a hospital-level information base for the HB-HTA data analysis management system. GWCMC had quite a lot of experience of HB-HTA and relevant data analysis modules had been included in their HIS systems. The module structure of the HB-HTA evaluation was divided into three stages: access and pre-selection research, first-level evaluation (objective evaluation), and second-level evaluation (subjective evaluation). Only technologies supported by relatively good-quality evidence passed the “access and pre-selection research” phase to enter the next evaluation phases. The first- and second-level assessments represented the objective and subjective parts, respectively.

After the evaluation, the hospital made recommendations on purchase and usage according to the HB-HTA scoring model’s scores. The hospital had 13 brands of sutures with an annual purchase volume of approximately 21 million units, and the product price differences were as high as 80 percent. There were 50 suture types, including 21 absorbable and 29 nonabsorbable/ordinary suture types. The annual procurement of sutures was approximately 21 million RMB: 17 million RMB for absorbable sutures and 4 million RMB for non-absorbable sutures. Details of the HB-HTA evaluation were not included in the submitted report, but they reported that based on the HB-HTA evaluation outcomes, the amount of absorbable sutures purchased could be reduced from 33 batches to 9, and most could be replaced by non-absorbable sutures. This recommendation would meet the clinical use without affecting clinical outcomes and greatly reduce the hospital’s operating costs. They estimated that the procurement cost of sutures could be reduced by more than 1 million RMB per year.

#### Lessons Learned from this Case

GWCMC made achievements in the following aspects by carrying out a pilot HB-HTA on sutures. First, the hospital reduced the number of suture brands significantly, from 13 to 4 brands, which also helped to reduce management costs. Second, the HB-HTA results suggested that more expensive absorbable sutures were not necessary in most clinical cases, thereby reducing the cost of sutures. In addition, the evaluation results were used as a basis for price negotiations with suture manufacturers and helped to lower prices. Third, it also helped to reduce the procurement workload.

### Case 4: HB-HTA of Transcatheter Aortic Valve Replacement in the QILU Hospital of Shandong University

#### Background of Hospital’s HB-HTA Project

The QILU Hospital of Shandong University has 5,300 beds and is the largest third-tier public hospital in Shandong Province, one of the largest provinces in China. For the HB-HTA pilot trial, the hospital assessed the transcatheter aortic valve replacement (TAVR) procedure. TAVR, the procedure of interest, is a new minimally invasive treatment technology that mainly uses the femoral and iliac arteries and other vessels as an approach to deliver the artificial aortic valve to the aortic valve ring through a catheter to complete the replacement of the aortic valve functionally. The comparator was a mechanical/biological surgical aortic valve replacement (SAVR). SAVR is a traditional procedure. The difference between TAVR and SAVR is that SAVR requires the cutting of the sternum, so the trauma is relatively large. Therefore, the surgery risk is high in older patients with poor basic physical condition or other complications, and postoperative recovery is slow. Relevant studies have shown that for patients with severe aortic stenosis in whom surgery is contraindicated, the one-year all-cause mortality rate after TAVR is lower than that after a conservative treatment; in other words, TAVR is superior to conservative treatments and SAVR (17). A comprehensive assessment was conducted involving safety, effectiveness, health economics, and other aspects of TAVR through a comparison with SAVR.

#### HB-HTA Process and Results

QILU Hospital had not previously carried out any HTA at the hospital level. In this pilot study, the project manager first established a multidisciplinary team called the TAVR-HTA team. The team consisted of a pilot project manager, who was also in charge of the hospital statistics team, a trained clinician, a surgeon who carried out TAVR at QILU Hospital, a nurse, and the pilot project manager’s assistant. The team first reviewed the available literature and concluded that no strong clinical evidence was available for patients with aortic valve stenosis in China; therefore, the team decided to collect hospital-based data. In addition to data extraction from the HIS, relevant individuals were interviewed to collect additional information. HTA was then carried out on a step-by-step basis using the template introduced by the authors’ institute and the European AdHopHTA.

The team collected RWD from the HB-HTA pilot study. RWD were sourced from the records of patients diagnosed with aortic stenosis and discharged from the hospital from September 1, 2017, to November 30, 2018. Data were collected from two groups of patients with aortic valve stenosis. The case group comprised patients who had TAVR, while the control group consisted of patients who had SAVR. The number of patients with aortic valve stenosis in the two groups varied, with a large number of patients with aortic valve stenosis in the control group. The team reduced the size of the control group by matching the two groups of patients with aortic valve stenosis according to age and sex and by setting up a 1:2 ratio (which was determined to be appropriate by the team).

Between September 2017 and November 2018, the hospital completed 10 TAVR procedures. Because one patient was a special case (due to the presence of other diseases) and one patient had not been discharged at the time of this HB-HTA project, eight patients were included in this study, including 3 male patients and 5 female patients. The mean patient age was 73.50 years old. The SAVR control group comprised 16 patients with an average age of 70.88 years. Although the aim was to match the two groups according to age, the age difference between the two real-world groups was statistically significant (*t* = 64.73, *P* < 0.0001), because the TAVR group included more older adults. This reflects the reality that older adults may have more complications and therefore may require more innovative approaches that are much less intrusive.

The safety, effectiveness, and economic aspects were also evaluated. In terms of safety, according to the preoperative evaluation, the average death risk of the eight patients in the TAVR group was 7.55 percent, which was worse than that in the SAVR group. Reviewing the postoperative complications, two patients in the TAVR group had postoperative complications, one patient had a permanent pacemaker implanted due to an atrioventricular block, and one patient had low cardiac output, respiratory failure, and renal failure. Only two postoperative complications occurred in the SAVR group. In terms of effectiveness, the main indicators of each case in the TAVR group before and after surgery were compared, indicating that the patient indicators changed significantly after surgery. Compared with the SAVR group, disease symptoms improved significantly. In terms of costs, only hospitalization expenses were collected, as costs occurred while the patients were in the hospitals. The average cost in the TAVR group was 14,300 RMB/hospitalization day and in the SAVR group it was 5,600 RMB/hospitalization day.

#### Lessons Learned from this Case

Although 20 years have passed since the world’s first human TAVR operation in 2002, TAVR is still a relatively new medical technology that needs to be developed and improved. Limited data are available on the development of an artificial valve delivery system with a smaller outer circumference, expansion of indications, control of complications, and adverse reactions. There is a lack of relevant systematic evaluations, especially of data that are essential for a health economics evaluation. The advantage of the HB-HTA is that the team can always collect RWD on the technology of interest from their own hospital. The QILU Hospital of Shandong University carried out the TAVR evaluation, relying heavily on technical support from the HB-HTA Pilots Organizing Center, NCMSA. The hospital HB-HTA team’s confidence grew with the development of the TAVR evaluation. This pilot study highlighted the importance of providing technical assistance to groups with no prior experience in HTA.

### Case 5: HB-HTA of Haploidentical Hematopoietic Stem Cell Transplantation for Aplastic Anemia at the First Hospital of Jilin University

#### Background of the HB-HTA Project

Aplastic anemia (AA) is a group of bone marrow hematopoietic failure syndromes caused by multiple etiologies with a very poor prognosis. Current treatments can be broadly divided into supportive and definitive therapies including immunosuppressive therapy (IST) and hematopoietic stem cell transplantation (HSCT).

In the field of HSCT, haplo-HSCT is considered a cutting-edge medical technology that may cure AA (especially severe aplastic anemia, SAA). Recently, practical innovations in haplo-HSCT in China have yielded satisfactory results. This pilot study aimed to analyze the safety, efficacy, and economic aspects of haplo-HSCT for AA and compare them with conventional IST to determine whether haplo-HSCT is suitable as the first treatment choice for patients with AA.

#### HB-HTA of haplo-HSCT for AA

This technology was introduced by the medical director of the Department of Hematology of the hospital as a treatment option in addition to IST for patients with AA. In this pilot project, an HTA expert committee comprising experts from the management and clinical departments was established. The committee was project-managed by a specifically established short-term within-hospital HTA office staffed by one person from the Medical Affairs Department and one junior doctor from the Department of Hematology. All of the study’s data were obtained from the HIS and did not involve the participation of manufacturers such as drug companies and device vendors.

This team followed the suggested HTA process provided by the NCMSA. First, a literature review was conducted to gather the available published evidence on haplo-HSCT for AA treatment. They searched PubMed using the keywords “transplantation” or/and “aplastic anemia” and found 747 papers, of which 60 papers on haplo-HSCT published after 2010 were selected and reviewed thoroughly. Second, the team summarized relevant assessment reports from other hospitals, academic institutions, and organizations in China and abroad. Subsequently, the team reviewed the AA guidelines for China and several other countries. The results of the literature review showed that haplo-HSCT is increasingly recognized as a cure for patients with IST failure or refractory SAA due to its clinical safety and effectiveness, and more importantly, its affordability. Haplo-HSCT is believed to have the potential to be a first-line treatment option for AA in the absence of HLA-identical sibling donors or matched unrelated donors; however, in practice, the procedure is still being explored.

Therefore, the HTA office decided to use RWD and to conduct a retrospective study of patients diagnosed with SAA and who received full treatment at the First Hospital of Jilin University from 2010 to 2019 and collected data from 27 patients treated with haplo-HSCT and 38 patients treated with IST for analysis.

Although the pilot project has not been fully completed due to a delay caused by staff redeployment, an evaluation framework has been determined. Overall survival (OS) was selected as the main index of efficacy. In terms of safety, the frequency and severity of rejection and postoperative complications arising from patients will be compared, as well as the mortality rate. The possible cost to the hospital and patients after the adoption of the new technology will also be calculated. Moreover, hospital believes that the introduction of cutting-edge technology plays an important role in the construction of hospital disciplines, the development of scientific research, and the improvement of social reputation.

#### Experiences and Lessons Learned from this Case

HB-HTA was not considered an essential step when clinicians and hospital managers made decisions about the implementation of new technologies in China, and the lack of persistence in applying HB-HTA in hospital decision making may be a common problem. The key may be the hospital management team’s determination to use HTA and consider its results in decision making. At the same time, besides establishing a long-term HTA department at an organizational level, it is more important to have professional staff with HTA knowledge and skills. It is also important to establish close communication between the management and clinical departments to facilitate the assessment process.

### Case 6: HTA of Fecal Flora Transplantation in People’s Hospital of Tianjin

The HB-HTA case at the People’s Hospital of Tianjin aimed to evaluate medical technology and fecal flora transplantation, which is still an innovative approach being explored in China.

#### Background of Hospital’s HB-HTA Project

Tianjin People’s Hospital is the largest public hospital in China. Anorectal surgery, one of the top-ranking specialties, was ranked best in the 2019 annual hospital evaluation in China. Tianjin People’s Hospital decided to carry out an HTA for the new clinical technology of fecal microbiota transplantation (FMT) for the treatment of slow transit constipation (STC). STC is a subtype of functional constipation (FC). Some bacteria in the intestine produce short-chain fatty acids and methane through the decomposition and digestion of dietary fiber, which are strong intestinal dynamic stimulants, whereas patients with slow-transit constipation lack these bacteria. FMT involves transplanting functional bacteria from the feces of healthy people into the intestines of patients to regulate the imbalance of intestinal bacteria. It is designed to rebuild the intestinal microecosystem and facilitate the treatment of diseases in and out of the intestine.

#### HB-HTA and Management Process

**Figure 2.**
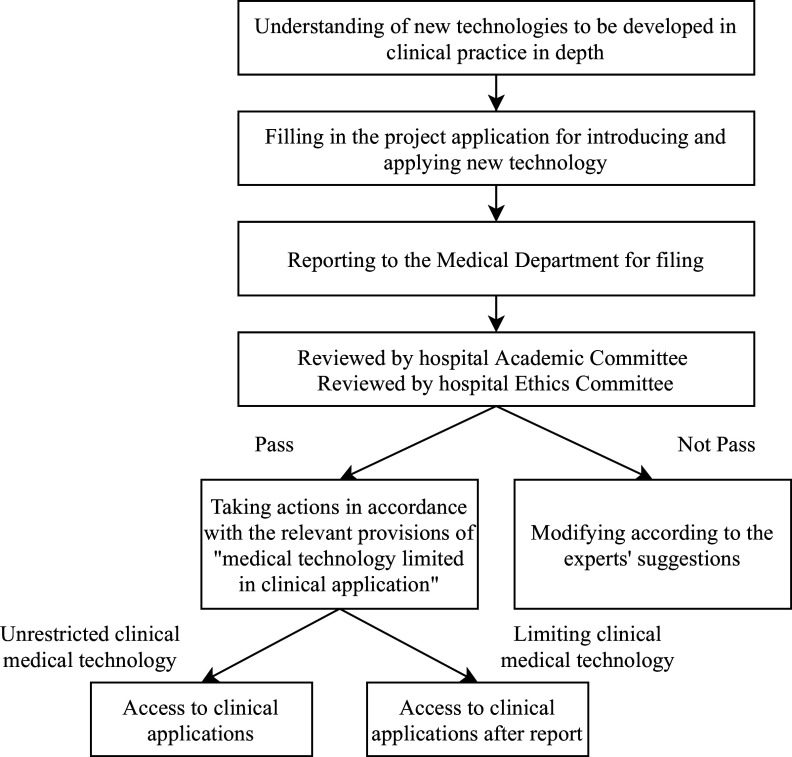
Management flow chart of new technology and new project of Tianjin People’s Hospital for hospital-based health technology assessment (HB-HTA).

The HB-HTA of the FMT project for the treatment of STC is reflected in the hospital’s new clinical technology management process (Figure 2). However, the project did not conduct an economic analysis of the technology and did not analyze the cost-effectiveness of the technology input and output, including medical resource saving, health efficiency improvement, and health status improvement. Only a clinical evidence review was carried out to determine the effectiveness of FMT compared to the standard treatment, including a literature review and an observational study to review and compare the clinical effectiveness in patients who received alternative treatments.

#### Lessons Learned from this Case

It was obvious from the HTA report submitted to the national institute that initiated the pilots that this project focused on clinical and ethical evaluation and did not follow the HTA process. It was the only pilot team that had a doctor as the team leader, and the doctor had very limited time to conduct the assessment. Thus, the skill sets of an HTA team leader and the entire team must include HTA and health economics. Therefore, it is important that all relevant participants involved in an HTA project are properly trained, especially those who perform the evaluation. In addition, there should be a proper prioritizing phase in which projects with strong clinical evidence and clear decisions should be determined as not requiring an HTA.

### Case 7: HB-HTA of a Management Model for Ventilators in the China–Japan Friendship Hospital

#### Background of the Hospital’s HB-HTA Project

The medical equipment referred to in this project was ventilators that can support a patient’s breathing, either partially or completely. The China-Japan Friendship Hospital currently has 1,610 beds and more than 300 ventilators. In 2017, the average usage of a ventilator was more than 10 hr per day; 53 clinical units in the hospital used ventilators, and the total duration of ventilator usage exceeded 300,000 hr per day. In this hospital, the management of the ventilators had become particularly difficult owing to frequent equipment lending among the wards of various departments. An analysis of the usage of the ventilators indicated that there were always some ventilators available, but the cross-department lending of ventilators had become problematic. Consequently, there had been procurement requests for ventilators from different departments over the last 5 years.

Therefore, the China-Japan Friendship Hospital was looking for a method of self-help within the hospital’s ventilator-sharing system. A new system was proposed by the hospital equipment management team based on an instant response system for ventilator usage data and Internet of Things (IOT) technology for the sharing and deployment of medical equipment. The hospital started the information management of ventilators based on IOT in 2017, and now by using a mobile APP management team can remotely monitor the status of ventilators by analyzing ventilator real-time data, tracing equipment maintenance, and collecting and analyzing quality control data automatically. Therefore, the operational model of self-help shared ventilators based on IOT was selected for evaluation in an HB-HTA because the management was uncertain about the system.

#### HB-HTA for Self-Help Shared Ventilator Allocation based on IOT

In the pilot project, a team consisting of four staff members from the Department of Healthcare Technology was formed. The team’s members had no prior HTA experience. The production of HTA included the assessment of operating efficiencies, clinical safety, and effectiveness of project implementation. As part of the HTA, additional information on the potential hazards, economic characteristics, and social effects of the technology was collected through communication with the heads of relevant clinic departments.

The research team conducted a literature review; however, no relevant studies were identified. Therefore, a pilot run of the within-hospital ventilator sharing system was carried out for 5 months, which included an assessment of safety, effectiveness, and cost-effectiveness. The hospital also conducted an information management project for medical equipment. The hardware and APP of the IOT sharing project have been developed on this basis. The development and operation costs of the hardware collector and APP software for equipment sharing were less than 300,000 RMB. During project implementation, activity-based costing was used to calculate the unit-time operating cost of the ventilator. The results indicated that the fixed cost per unit time of the ventilator was approximately 4 RMB/h. The benefits included the monetary benefits of savings gained from not purchasing new ventilators as a result of sharing and also charges made on the number of hours of ventilators usage, and the benefits from providing ventilator services for patients in need, estimated by the frequency of lending and the hours of ventilators usage. Sensitivity or scenario analyses were not conducted to address uncertainties related to the evidence owing to a lack of comprehensive knowledge of HTA methods.

The data analysis showed that 7 months after the implementation of the sharing system, 39 clinical units used the shared ventilators for a total of approximately 20,000 hr, which is approximately 1/10 of the total hours of all ventilator usage. Improvements in patient experience were recognized by both patients and staff, and cases of machine failure were similar to those before. Using the self-help shared process saved about 420,000 RMB in 2018. In 2018, only four new ventilators were purchased for the new pediatric intensive care unit (PICU), which saved about 2.5 million RMB for equipment purchase funds. In addition, after the implementation of the sharing system, the hospital deployment center no longer needed special personnel on call 24/7, which reduced the human cost for the hospital.

#### Lessons Learned from the Case of HB-HTA of Innovative Management Model of Medical Equipment

First, the HB-HTA demonstrates that this type of assessment can be applied to evaluate health technology in a broad sense and can be successfully applied to evaluate a management model rather than just a drug or device. Although there was a lack of supporting material and data, the China–Japan Friendship Hospital team completed the evaluation using information from interviews with clinical experts and a pilot run of the system. Furthermore, compared with drugs or procedures, there are fewer health technology assessments of management models (18). Second, data collection is crucial for a medical equipment management model. None of the evaluations would be convincing if relevant data could not be collected within the hospital equipment monitoring system; in this case, if ventilators could not be monitored, ventilator sharing would not be possible. Finally, interested parties participated in the HB-HTA to promote the transformation of health technology from an administrative to an evidence-based decision making tool, and to avoid evidence-centered administrative decision making as much as possible. However, hospital leadership plays a significant role in HB-HTA. Without the strong support of the hospital leadership, in-depth HB-HTA cannot thrive.

More outcomes of the seven pilot projects are shown in Table 3.

**Table 3.** Checklist of HTA as outcomes of the seven pilot projects

		Case 1	Case 2	Case 3	Case 4	Case 5	Case 6	Case 7
Summary	Summary of effects							
Basic information	Proposer of the technology	Medical consumables management department of hospital	Medical consumables management department of hospital	Medical consumables management department of hospital	Surgical department	Surgical department	Clinical department	Medical equipment management department
	Other parties/stakeholders	Operational department, clinical specialists, manufacturers	Operational department, clinical specialists, manufacturers	Operational department, clinical specialists, manufacturers	Operational department, clinical specialists, manufacturers	Operational department, clinical specialists, manufacturers	Medical technology clinical application management committee, Hospital ethics committee, clinical specialists	All in-need-of-ventilator clinic departments, information department, financial department, IT companies
	Some possible conflicts of interest	Not stated	Not stated	Not stated	Not stated	Not stated	Not stated	Not stated
	Report review	External reviewed	External reviewed	×	×	×	×	×
	Goal and scope of the report	√	√	√	√	√	√	√
General methodological aspects and reporting	Review of relevant literature	34 papers from PUBMED, CNKI, WANFANG DATE and NICE						Searched and found no relevant literature
	Additional material/data	Hospital real-world data	Hospital real-world data	Hospital real-world data	Hospital real-world data	Hospital real-world data	Expert opinions	Hospital real-world data
	Quality of information/data/studies	High	High	Median	Median	Median	Median	Low
	List of references	√	√	√	√	√	√	×
	Ongoing studies of the effect of the proposal	Within-hospital reviews and published articles	Within-hospital reviews and extensive published articles	Within-hospital reviews	Within-hospital reviews	Within-hospital reviews	Within-hospital reviews	Within-hospital reviews
Results within domains	Clinical effectiveness	√ (S2-PD form was used to measure outcome in trials but not possible in real-world as follow-up need to be 1–2 years longer)	√	√	√	√	√	√
	Patient safety	√	√	√	√	√	√	(i) Safety evaluation of ventilator in clinical use; (ii) Evaluation of effective disinfection after ventilator use; (iii) Reliability evaluation of ventilator operation
	Economic aspects	Costs and fees(trauma group further strictly controlled the indications, The proportion of human acellular nerve graft decreased, and average price for the use of similar materials dropped from 12 996 yuan to 11 189 yuan)	Costs and fees	Costs and fees	Costs and fees	Costs and fees	Lack of economic assessment	(i) Cost of project implementation; (ii) Benefits from the implementation of the project
	Organizational aspects	×	Not stated	Not stated	Not stated	Not stated	√	√ (Save human resources)
	Patients’ perceptions	√	√	√	√	√	Stated a need from patients	×
	Strategic aspects	×	Not stated	Not stated	Not stated	Not stated	Consolidate technology leadership	Makes a meaningful exploration on the information management of smart hospital under the condition of 5G in the future
	Other potentially important aspects	×	×	×	×	×	×	×
Discussion, conclusion, and recommendations	Discussion of uncertainties	√	√	√	√	√	√	√
	Implementation in other hospitals	×	Not stated	Not stated	Not stated	Not stated	√	×
	Recommendation of other relevant national/international institutions or organizations	×	Not stated	Not stated	Not stated	Not stated	√	×
	Recommendations based on the assessment	√	Not stated	Not stated	Not stated	Not stated	√	√
	Suggestions for further actions	√	√	√	√	√	√	√

HTA, health technology assessment.

## Discussion

### The Value of HB-HTA

The experiences of the seven hospitals with the production and use of HB-HTA indicate that hospital managements see the value of the HTAs in terms of four aspects:

First, HTA follows the rules of evidence-based medicine and is comprehensive and systematic, so it helps decision-makers consider all aspects, which is especially important when something unexpected is revealed.

Second, hospital managers have greater confidence in their decisions and investments when using the HB-HTA as the basis for their decision making.

Third, a clear evaluation process makes the management of new technology access applications easier to tackle down. The Chief of Medical Services in one of the pilots said during an interview that the number of applications dropped by a third after they announced the new process, and these applications lacked evidence or potential benefits. This was expected because the previous application procedure was not very strict and many applications were submitted without sufficient evidence. The new procedure required applicants to strictly follow the established procedure to write application reports with supporting materials.

Fourth, The HB-HTA helps hospitals to control costs. Health reform in China has started to enforce the implementation of DRGs (a payment method based on disease-related groups), which has forced hospital managers in all public hospitals to focus increasingly on the details of services provided to avoid losing money in the new form of financing. Applying the HB-HTA could help to determine the costs of providing a service and the reimbursement in the form of DRG.

### Experience and Challenges of the HB-HTA Pilot Project in China

China’s HB-HTA pilots were initiated by the authors’ institute to promote HB-HTA in public hospitals. The need for the development of HB-HTA is closely related to the decentralization of China’s medical system, as health reforms have given hospitals more autonomy in decision making regarding access, procurement, and management of health technologies. HB-HTA methods are relatively “mature” and include efficacy, safety, organizational, and economic evaluations, as well as ethical assessments. Different hospital managers may have different needs for the information that affects their decision making (19). Currently, the main contribution of the HB-HTA pilots is to make the tool accessible to hospital managers in China. In addition, the goal of the pilots was to explore and improve the best-fit HB-HTA settings in China. The evaluation process and evaluation methods need to be further explored and improved, gradually institutionalizing the use of HB-HTA in decision making for all top hospitals.

Currently, the challenge of HB-HTA in China is that the understanding of health technology assessment is too insufficient for hospitals to take actions to apply this tool. That said, the first HTA research institution was founded in 1994, and the National Health Commission of China established the National Comprehensive Assessment Center for Drugs and Health Technology in December 2018 to support HTA skills in technology admission, pricing, medical insurance reimbursement, and so forth for national-level decision making. However, very few hospitals were familiar with the terms HTA and HB-HTA at the beginning of the first pilot. Hospital managers often make administrative decisions regarding health technology investment or disinvestment without proper evidence. Hospitals intending to apply HB-HTA are initially faced with a lack of funds and/or professional staff. Currently, the ability to implement HB-HTA is limited. However, the pilots were successful in promoting HB-HTA, as evidenced by the fact that more hospitals joined the second cohort.

Nevertheless, it needs to be recognized that although all seven hospitals completed their HB-HTA trial projects and some finished with a proper HTA report, many problems were reported. First, hospitals with no experience need guidance, but there is a lack of a national assessment process and methodology guidelines. Second, there is a lack of skills to conduct economic analyses, as most hospitals do not recruit graduates familiar with HTA. Third, in general, there is a lack of motivation from hospital managers to initiate HB-HTA. However, with the implementation of DRGs, HB-HTA is rapidly gaining popularity. Last, the follow-up evaluation of decisions based on HB-HTA was absent or weak, and a lack of feedback makes it even more difficult to promote HB-HTA.

## Conclusions

To allow HB-HTA to play a greater role in health policy, a transparent process involving all relevant stakeholders needs to be promoted. The literature and information gathered from the pilots and case studies show that the participation of all relevant stakeholders is very important for HB-HTA. This phenomenon has also been observed in other countries (20). The emphasis on the participation of stakeholders during an HTA so that the participation process involves not only decision-makers and a multidisciplinary team inside the hospital but also patients and manufacturers outside the hospital (21) needs to be promoted. Process transparency is an important foundation of HB-HTA credibility; the quality and timeliness of reports also affect the adoption of HTA reports (22). China’s HB-HTA also needs to improve its governance structure and actively invite stakeholders to participate in the process to ensure that it is open and transparent to improve the quality and credibility of HB-HTA reports.

The interviews conducted for the seven cases described above show that management support is generally considered important to ensure the success of HB-HTA. However, there is also a risk associated with the involvement of management – what should be an evidence-based practice could become an eminence-based practice and just a way to justify what managers really want to buy or change. This is a dilemma that HB-HTA producers need to focus on and consider in the production and use of HTA as a basis for decision making. This seems to be entirely generic and true for any country or region. However, owing to the higher degree of centralization in China’s healthcare system, the decision making influence of health system leaders and hospital managers on the implementation of HB-HTA in hospitals is also greater. For example, the fear of being held accountable could make many hospital managers unwilling to attempt something new under the centralized system and content with the status quo.

Second, after the completion of a HB-HTA pilot, the team should summarize and propagate their experience to support HB-HTA in their own hospitals and inspire others. However, owing to the large number of hospitals in China, it is impossible and unnecessary for all hospitals to conduct such activities. We suggest that China should support the establishment of HB-HTA cooperation networks – in the footsteps of health reform – and the construction of medical alliances to form regional HB-HTA networks and nationally specialized HB-HTA networks (such as an HB-HTA network in pediatrics), so that more hospitals can benefit from HB-HTA. Real-world hospital data can not only fill gaps in published evidence, but can also improve the generalizability of the evidence to a local setting (23).

Third, the real use of the HB-HTA is to support health policy decisions within hospitals. Therefore, it is important to translate scientific evidence into policy during the development of HB-HTA (24). The introduction of HB-HTA as a decision-support tool for municipalities was insufficient and should have been supplemented with a strategy to secure local political and managerial support and willingness (25). Thus, the implementation of HTA is not just as a question of how to increase the use of evidence in decision making but also a matter of reforming local decision-making processes. Therefore, the future development and direction of the HB-HTA program should promote the application of HTA results in real decision making in hospitals.

Finally, the quality of HB-HTA is the key to its successful implementation. Therefore, it is important to have full support in two aspects: the source of evidence and the staff who produce the HTA. It is essential for hospitals to have full-text access to major scientific journals so that staff have clear access to all relevant studies. We also recommend that HB-HTAs be collected in a national database. Progress has been made in the national institute that initiated HB-HTA pilots to provide a web platform. We highly recommend that in the education sector, HTA and evidence-based practice (EBP) should be set as compulsory courses for medical students at Chinese universities and that HTA and EBP training courses should be provided to those working in hospitals as regular capacity-improvement schemes.
